# Regulation of Sumo mRNA during Endoplasmic Reticulum Stress

**DOI:** 10.1371/journal.pone.0075723

**Published:** 2013-09-18

**Authors:** Kristin A. Moore, Joshua J. Plant, Deepika Gaddam, Jonathan Craft, Julie Hollien

**Affiliations:** Department of Biology and the Center for Cell and Genome Science, University of Utah, Salt Lake City, Utah, United States of America; St. Jude Children's Hospital, United States of America

## Abstract

The unfolded protein response (UPR) is a collection of pathways that maintains the protein secretory pathway during the many physiological and pathological conditions that cause stress in the endoplasmic reticulum (ER). The UPR is mediated in part by Ire1, an ER transmembrane kinase and endoribonuclease that is activated when misfolded proteins accumulate in the ER. Ire1's nuclease initiates the cytosolic splicing of the mRNA encoding X-box binding protein (Xbp1), a potent transcription factor that then upregulates genes responsible for restoring ER function. This same nuclease is responsible for the degradation of many other mRNAs that are localized to the ER, through Regulated Ire1 Dependent Decay (RIDD). Here we show that Smt3, a homolog of small ubiquitin-like modifier (sumo), is a non-canonical RIDD target in *Drosophila* S2 cells. Unlike other RIDD targets, the sumo transcript does not stably associate with the ER membrane, but instead relies on an Xbp1-like stem loop and a second UPR mediator, Perk, for its degradation during stress.

## Introduction

The flux of proteins through the secretory pathway varies extensively among cell types and different pathological and physiological conditions. As demand for secreted proteins changes, so do the systems within the endoplasmic reticulum (ER) that are responsible for protein folding and processing. ER stress results when accumulation of unfolded proteins overcomes the folding capacity of the ER. In metazoans, this situation is sensed by three main classes of ER transmembrane proteins- Ire1, Perk, and Atf6- which together mediate the numerous changes in gene expression that define the unfolded protein response (UPR) [1], [2]. This response is essential for normal development in mammals and is thought to impact several diseases, including diabetes, cancer, and neurodegenerative disorders [3].

The UPR has broad effects on transcription, translation, and mRNA decay during ER stress. Translational regulation is mediated largely by Perk, which dimerizes during ER stress and is activated through autophosphorylation [4], [5]. Perk phosphorylates the translation initiation factor eIF2α, thereby inhibiting cap-dependent translation of most transcripts [6], [7]. However, transcripts containing upstream open reading frames (uORFs), such as the basic-leucine zipper (b-zip) transcription factor Atf4, are selectively translated in these conditions and thus their expression increases during ER stress [8]. Ire1, a second mediator of the UPR, oligomerizes during stress, leading to activation of its cytosolic kinase and endoribonuclease domains [9], [10], [11]. Ire1 specifically cleaves the mRNA encoding X-box binding protein (Xbp1), directly leading to the cytosolic splicing and translation of this b-zip transcription factor [12], [13]. Along with Atf4 and Atf6 (a third b-zip transcription factor activated by proteolysis during ER stress [14]), Xbp1 transcriptionally upregulates many genes encoding ER-specific protein folding chaperones and other proteins that function in the secretory pathway [15], [16]. Ire1 is also necessary for cleavage of many other mRNAs, initiating their degradation through Regulated Ire1 Dependent Decay (RIDD) [17], [18], [19].

Although much is known about the mechanism of Xbp1 splicing, the features of mRNAs that identify them as RIDD targets have been more elusive. In *Drosophila melanogaster* cells, localization to the ER membrane appears to be the major factor in targeting mRNAs to this pathway; ER-targeting signals are both necessary and sufficient for degradation by RIDD [17], [20], and there is a strong correlation between the extent of membrane association of a given mRNA and its degradation by RIDD during ER stress [20]. Conversely, cleavage site specificity does not appear to be important for RIDD targeting in *Drosophila* [20]. Based on gene ontology classifications, RIDD targets in mammals and *S. pombe* are enriched for mRNAs encoding secretory proteins, and therefore are presumed to be localized to the ER [18], [19], [21]. However, RNA localization does not appear to fully account for the specificity of RIDD in these organisms, suggesting that there are other targeting requirements. These requirements may include specific sequences such as the stem loop structures that define the cleavage sites in Xbp1 and are also enriched in mammalian RIDD targets [18], [19], [22].

Interestingly smt3, the *D. melanogaster* homolog of sumo, was identified in microarray experiments as a potential RIDD target [17], despite lacking any recognizable sequence elements that would target it to the ER. This observation led us to hypothesize that the sumo transcript may rely on different mechanisms for degradation compared to the majority of RIDD targets in flies. Here we demonstrate that the mRNA encoding sumo is a non-canonical RIDD target and depends on both an Xbp1-like stem loop structure and Perk for its degradation during ER stress.

## Results

### The mRNA encoding sumo is a non-canonical RIDD target

**Figure 1:**
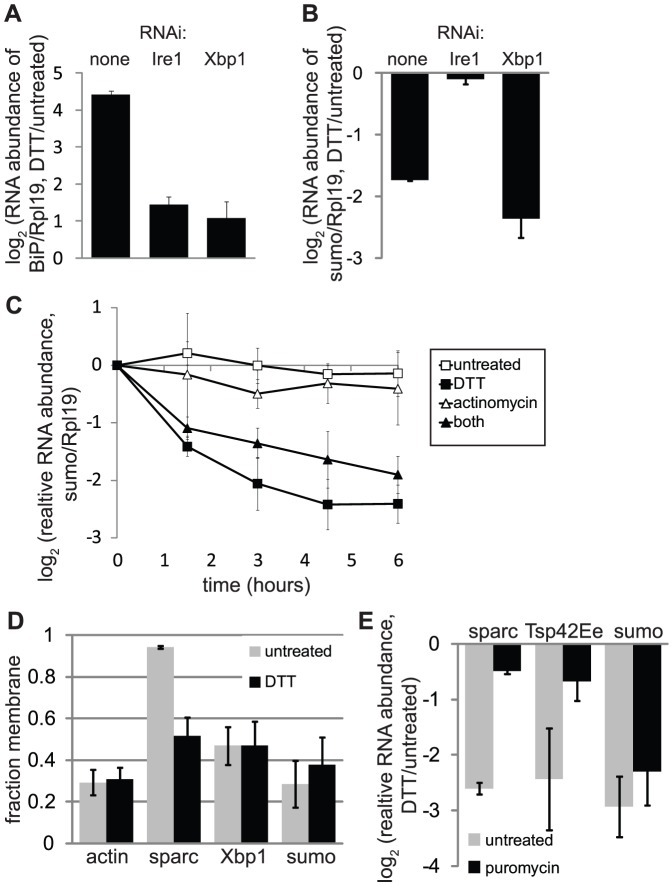
Sumo mRNA is a non-canonical RIDD target. For all panels, we measured relative RNA abundance by qPCR; shown are the averages and SDs of 3-4 independent experiments. Except for the fractionation in panel D, we normalized all mRNA abundance measurements to the housekeeping control Rpl19. A-B. Relative mRNA levels of BiP (panel A) and sumo (panel B) in mock-treated and Ire1- or Xbp1-depleted *Drosophila* S2 cells incubated in the absence and presence of ER stress (2 mM DTT, 4.5 hours). Xbp1 transcript levels in the Xbp1 RNAi-treated cells were 13.5% +/− 0.9% of the levels in control cells, as measured by qPCR. C. Timecourse of sumo mRNA levels in S2 cells treated with or without actinomycin D (1 µg/mL) to block transcription and DTT (2 mM) to induce ER stress. D. Fraction membrane (membrane/total) for mRNAs from S2 cells treated with and without DTT (2 mM, 20 min). We separated cytosolic and membrane RNAs using detergent extraction (see Materials and Methods). E. Relative mRNA levels in cells treated with or without 35 µM puromycin (added 10 min prior to stress) and DTT (2 mM, 4 hrs).

We previously observed by microarray that the relative amount of the sumo (smt3, CG4494) transcript decreases during ER stress in *D. melanogaster* S2 cells, in an Ire1-dependent but Xbp1-independent manner [17]. We confirmed this result here by quantitative real-time PCR (qPCR) (Figure 1A-B). Depletion of either Ire1 or Xbp1 by RNAi inhibited the upregulation of BiP, a major ER chaperone, during ER stress (Figure 1A). However, depletion of Ire1 but not Xbp1 blocked the downregulation of sumo mRNA (Figure 1B). To test whether this decrease was the result of mRNA decay, we treated S2 cells with actinomycin D (1 µg/mL) to block transcription and collected samples over time in the presence and absence of dithiothreitol (DTT, 2 mM), a reducing agent that strongly induces ER stress. Tunicamycin and thapsigargin, two other strong inducers of ER stress in mammalian cells, do not efficiently activate Ire1 in S2 cells [17], thus DTT was used to activate ER stress pathways in the following experiments. Sumo mRNA levels were stable in actinomycin-treated cells over six hours, but significantly decreased over time during ER stress (Figure 1C). Therefore, sumo is a RIDD target.

While ER localization appears to be necessary and sufficient to target mRNAs to RIDD in S2 cells [17], [20], sumo contains neither a signal sequence nor a transmembrane domain, and thus its mRNA cannot localize to the ER by conventional mechanisms. To determine experimentally whether this mRNA is localized to the ER through an alternative pathway, we used a previously-described detergent fractionation method [20], [23] to separate membrane-bound vs. cytosolic mRNAs from S2 cells. As predicted from its sequence and the known cytosolic/nuclear functions of the sumo protein, sumo mRNA was highly enriched in the cytosolic fraction, along with the mRNA encoding actin (Figure 1D). Its fractionation behavior did not change with ER stress (Figure 1D), although as we have previously found, the membrane-associated mRNA sparc became more digitonin-extractable during ER stress, perhaps due to the concurrent attenuation of translation [20]. Interestingly, Xbp1 mRNA, which is cleaved by Ire1 during stress, also did not strongly fractionate with the membrane (Figure 1D), suggesting that strong, stable association with the ER is not absolutely required for cleavage by Ire1.

To further test a possible role for ER localization in the degradation of sumo mRNA, we treated S2 cells with puromycin (35 µM), a translation elongation inhibitor that releases mRNAs from ribosomes and disrupts the ER localization of mRNAs that rely on translation-dependent mechanisms of membrane targeting. Degradation of the sumo transcript during ER stress was not significantly affected by puromycin treatment (Figure 1E). In contrast, other RIDD targets (sparc and Tsp42Ee) were no longer degraded in the presence of puromycin, most likely because the mRNAs were no longer associated with the ER. These results suggest that ribosome-dependent membrane localization is not necessary for RIDD targeting of sumo mRNA.

### An Xbp1-like stem loop is necessary and sufficient for targeting sumo mRNA to RIDD

**Figure 2:**
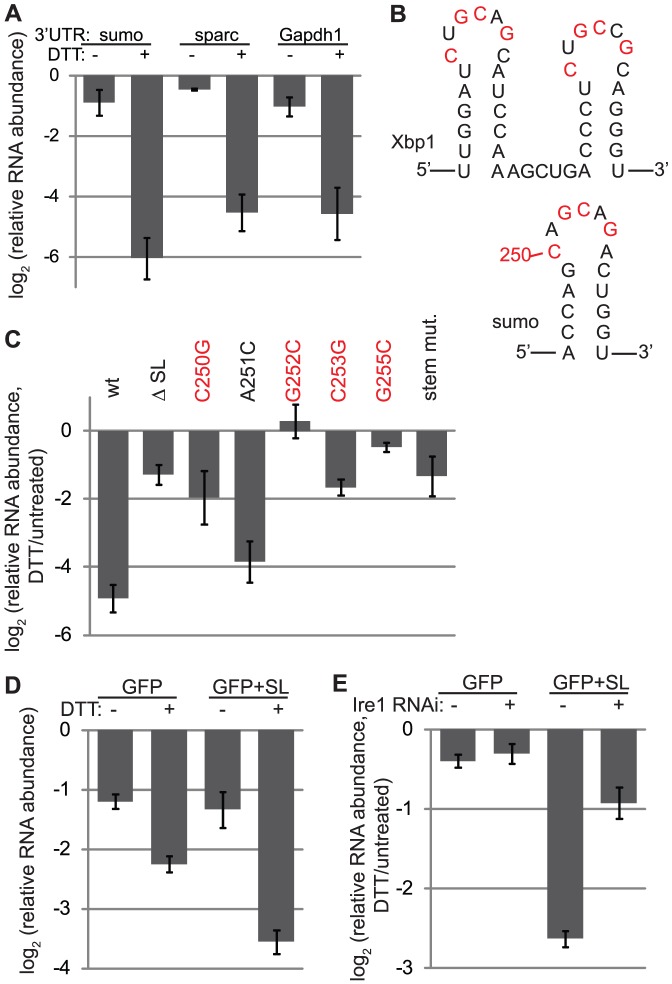
A stem loop sequence in the sumo mRNA is important for RIDD targeting. For panels A, C-E: plasmids expressing reporter mRNAs under the control of a copper-inducible promoter were stably transfected into S2 cells. After inducing expression, we removed the copper to stop transcription of reporter mRNAs, incubated cells in the presence and absence of ER stress (2 mM DTT, 5 hrs), and collected RNA samples. Relative RNA abundance was measured by qPCR and normalized to Rpl19. Shown are the averages and SDs of 3 (panels A, C) or 2 (panels D-E) independent experiments. A. Reporters expressing the coding sequence of sumo followed by various 3′UTRs. We normalized RNA levels to a control sample collected immediately before washing out the copper; thus RNA measurements reflect the amount of degradation after 5 hrs without copper. B. RNA sequences of sumo and Xbp1 from *D. melanogaster,* surrounding the stem loop structures discussed here. Highlighted in red are the loop nucleotides conserved in Xbp1 across species. Numbering in the sumo mRNA is relative to the translation start site. C. Reporters containing the sumo coding sequence and 3′ UTR, with various mutations. ΔSL =  deletion of nucleotides 244–270 in the coding sequence of sumo; stem mut. =  C257G/G259C/G260C. D. Reporters expressing GFP with the Gapdh1 3′UTR, with and without the stemloop sequence of sumo (nt 244–270). E. Degradation of reporters from D in untreated cells and those depleted of Ire1.

To examine the *cis* elements in the sumo transcript important for its degradation during ER stress, we used reporter plasmids expressing the coding sequence of sumo under the control of the copper-inducible metallothionein promoter. After inducing expression of reporter mRNAs in S2 cells with CuSO_4_, we removed the transcriptional inducer and monitored mRNA degradation in the presence and absence of ER stress. Although regulation of localization, translation, and degradation of mRNAs often relies on sequence elements within the 3′UTR, we found that replacing the sumo 3′UTR with that of sparc (an ER-localized RIDD target) or Gapdh1 (a cytosolic mRNA unaffected by ER stress) did not affect its targeting to RIDD (Figure 2A).

Further sequence analysis, however, revealed that the *D. melanogaster* sumo transcript contains a predicted stem loop near the end of its coding sequence that bears a striking similarity to the Xbp1 stem loop sequences that are cleaved by Ire1 (Figure 2B). Deletion of the 27 nucleotides surrounding this structure abolished ER stress-dependent degradation of the sumo mRNA reporter (Figure 2C). To probe this sequence more specifically, we made point mutants within the loop and the stem. Mutation of any of the 4 conserved bases within the 7-member loop [24] blocked ER stress-dependent degradation of sumo mRNA, whereas mutation of a non-conserved base in the loop had no effect (Figure 2C). Likewise, mutation of 3 nucleotides within the predicted stem structure also blocked degradation (Figure 2C).

To determine whether the Xbp1-like stem loop within the sumo transcript is sufficient for targeting an mRNA to RIDD, we used a reporter plasmid encoding GFP. The GFP mRNA alone is not a RIDD target ([20] and Figure 2D, E). However, addition of the 27 nucleotides surrounding the sumo stem loop to the 3′ end of the GFP coding sequence led to an ER stress-dependent increase in the degradation of GFP mRNA (Figure 2D). We then tested whether this degradation was Ire1 dependent by depleting Ire1 through RNAi. Degradation of the GFP mRNA alone was unaffected by Ire1 depletion, whereas the enhanced degradation of the GFP-stem loop mRNA seen during ER stress was inhibited by Ire1 depletion (Figure 2E). Thus, degradation of the GFP-stem loop mRNA occurs through RIDD.

### Sumo is not a strong RIDD target in mammalian cells

**Figure 3:**
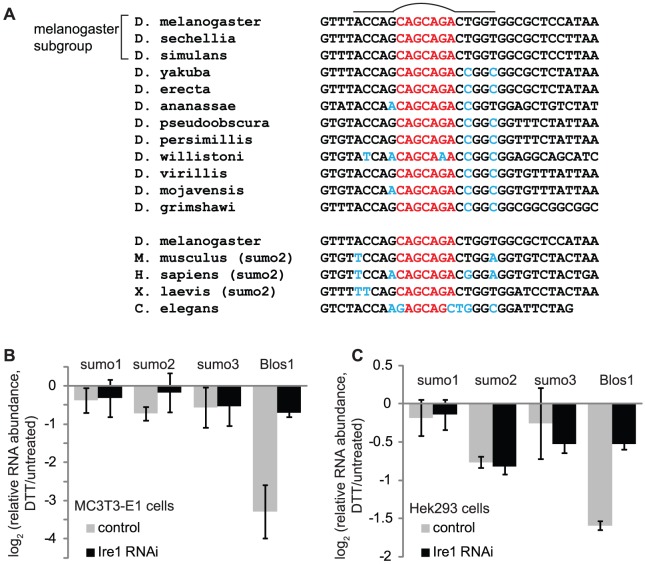
Sumo mRNA is not strongly affected by ER stress in mammalian cells. A. Conservation of the Ire1 site and surrounding region in sumo transcripts across species. The stem loop is indicated above the sequences and the region aligning with the loop from *D. melanogaster* sumo is shown in red. Deviations from the *D. melanogaster* sequence in the stem loop region are shown in blue. While most species have a conserved loop sequence, perfect basepairing in the stem is present only in the fly and mouse sequences. B-C. We either mock-treated (control) or used siRNA to deplete Ire1α from MC3T3-E1 mouse osteoblastic fibroblasts (panel B) or Hek293 human kidney cells (panel C). We then compared RNA levels in the presence and absence of DTT (2 mM, 4.5 hrs), by qPCR. Blos1, a RIDD target in mouse and humans, is shown as a control. Except for Blos1, the differences in mRNA levels between control and Ire1 siRNA-treated cells were not statistically significant. Shown are the averages and SDs for 2–3 independent experiments.

To determine whether regulation of sumo mRNA by RIDD is conserved, we searched for Xbp1-like stem loops in sumo transcripts of other organisms, using the criteria that an Ire1 site must contain a stem loop with at least 5 basepairs in the stem and exactly 7 nucleotides in the loop, and must contain the four conserved loop nucleotides depicted in Figure 2B. The Ire1 site was not widely conserved; even within *Drosophila*, we found Ire1 sites in the sumo transcripts of only 2 of the 11 species we examined, namely *D. sechellia* and *D. simulans*, the closest relatives to *D. melanogaster* (Figure 3A). We did not uncover any predicted Ire1 sites in the sumo transcripts for humans, *X. laevis*, or *C. elegans*.

Despite this lack of general conservation, we did find an Ire1 site in a mouse sumo transcript. Mice possess three sumo genes, in contrast to *D. melanogaster*, which has only one. While neither sumo1 nor sumo3 contains a predicted Ire1 site, sumo2 has a stem loop at exactly the same position, relative to the coding sequence, as the one in *D. melanogaster* sumo. The loop and first four basepairs of the stem are perfectly conserved between these two transcripts (Figure 3A).

To determine whether sumo2 is downregulated during ER stress in mouse cells, we treated mouse preosteoblast MC3T3-E1 cells with DTT (2 mM, 4 hrs) and measured mRNA levels for the mouse sumo homologs by qPCR (Figure 3B). These cells robustly degrade the RIDD target Blos1 in response to ER stress. Sumo2 displayed a very weak downregulation (p-value for untreated vs. DTT-treated = 0.08). Depletion of Ire1 by RNAi blocked the degradation of Blos1, and appeared to also affect sumo2 down-regulation; however the overall effect was weak and did not pass the standard p-value cutoff for statistical significance, using a paired t-test (p-value for dtt/untreated, control vs Ire1 RNAi = 0.2).

To account for potential variation in Ire1 site preferences and test the possibility of sumo regulation in human cells, we repeated the above experiments in HEK293 cells (Figure 3C). Treatment with DTT (2 mM, 4 hrs) resulted in a small but significant decrease in sumo2 mRNA levels (p-value = 0.02). This effect was not Ire1-dependent, consistent with the lack of predicted Ire1 sites in human sumo transcripts. Levels of sumo1 and 3 remained unchanged in both MC3T3-E1 and HEK293 cell types. These results suggest that while sumo is downregulated in mammals during ER stress, the effect is small and the mechanisms regulating sumo levels vary between organisms.

### RIDD of the sumo transcript is dependent on Perk

**Figure 4:**
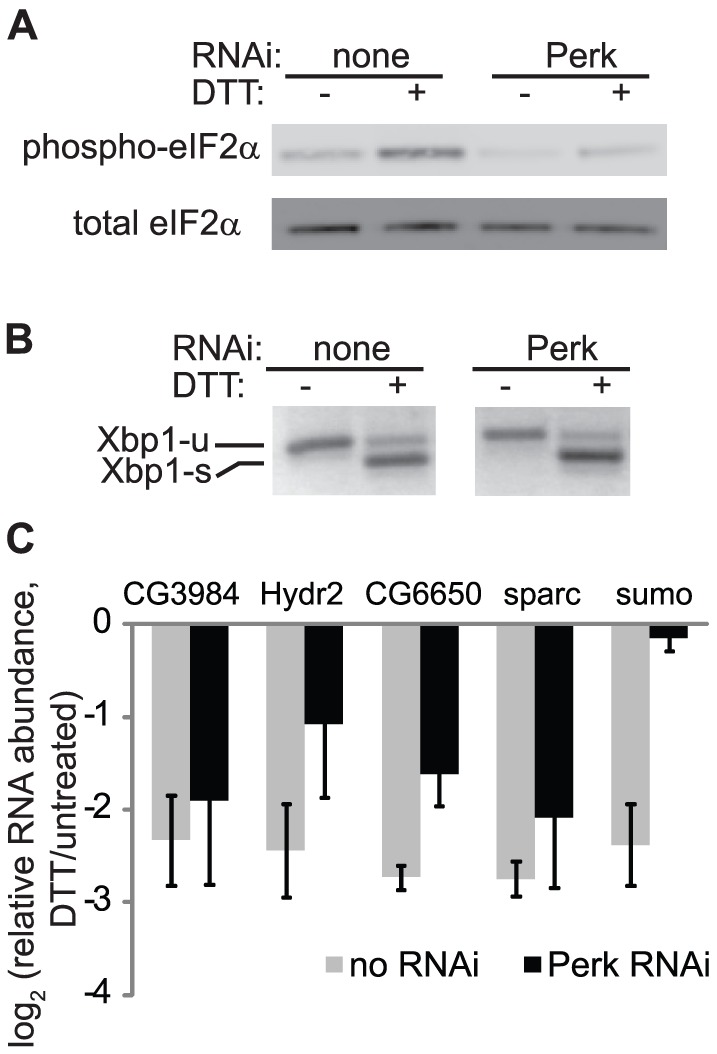
RIDD of sumo is dependent on Perk. We used RNAi to deplete S2 cells of Perk. A. Western blot showing the levels of phosphorylated and total eIF2α. B. Agarose gel showing the relative levels of unspliced and spliced Xbp1, amplified by reverse-transcription-PCR using primers surrounding the splice site. C. Relative mRNA levels in ER stress treated vs untreated cells, determined by qPCR. Panels A-B show representative data, panel C is the average and SD of 3 independent experiments.

During ER stress, Perk activation and phosphorylation of eIF2α result in an attenuation of translation, which can affect mRNA stability [25], [26]. To test whether Perk is important for degradation of sumo mRNA, we depleted Perk by RNAi and measured the relative abundance of endogenous sumo mRNA in the presence and absence of ER stress. Strikingly, degradation of the sumo transcript during ER stress was completely abolished in the absence of Perk (Figure 4C). Sumo mRNA levels in the absence of stress were not affected by Perk depletion (levels in Perk RNAi/control cells = 0.84, p = 0.5). Furthermore, Xbp1 splicing (Figure 4B) and degradation of RIDD targets CG3984, Hydr2, and sparc (Figure 4C) were largely unaffected, although degradation of CG6650, another RIDD target, was partially inhibited. Western blot analysis of the phosphorylation of eIF2α confirmed the efficient knockdown of Perk (Figure 4A), whose mRNA levels were reduced to 39% +/− 7% compared to controls, as measured by qPCR. These data suggest that Ire1-dependent degradation of sumo mRNA is particularly sensitive to Perk activity.

## Discussion

ER stress occurs in many physiological and pathological conditions, and the response to accumulation of misfolded proteins can determine cell fate. While much is known about the initiation and downstream effects of transcriptional regulation of mRNAs during the UPR, the features that target mRNAs to the RIDD pathway are less well understood. We previously found that in *Drosophila* S2 cells, ER localization is both necessary and sufficient for targeting mRNAs to RIDD [17], [20], while recognizable Ire1 cleavage sites are not predictors of RIDD targeting [20]. Furthermore, previous mutagenesis experiments found a distinct lack of specific sequence elements affecting degradation, other than ER-targeting signal sequences [17]. Here we demonstrate that the mRNA encoding sumo is an exception to these rules. Although the sumo transcript is degraded by RIDD, it is not stably associated with membranes. Degradation strongly depends on a specific *cis* element in the sumo coding sequence, comprised of a stem loop structure very similar to the conserved Ire1 recognition sites in Xbp1, and mutagenesis of the conserved bases within this stem loop inhibits degradation of the transcript. This parallels mutagenesis experiments showing that these same conserved bases within the Hac1 (the Xbp1 homolog in yeast) and Xbp1 stem loops are important for cleavage and splicing [27], [28], [29].

This distinct targeting mechanism suggests that downregulation of sumo serves a different function during ER stress, compared to the degradation of other RIDD targets, which may relieve stress by reducing the protein folding load on the ER. The sumo protein covalently modifies many target proteins, often affecting protein localization and activity [30]. Interestingly, the spliced version of mouse Xbp1 can be SUMOylated, leading to a decrease in transcriptional activation of target genes [31], [32]. Thus during ER stress, degradation of sumo mRNA may enhance UPR signaling. However, the stem loop structure shown here to be critical for RIDD of sumo does not appear to be widely conserved beyond *D. melanogaster*, and sumo mRNA is only very weakly down-regulated in the mammalian cells we have tested.

Sumo regulation is highly sensitive to Perk, as its degradation is completely abolished when Perk is depleted. This is in contrast to canonical *Drosophila* RIDD targets, which are only mildly sensitive to Perk depletion (Figure 4C and [17]). The mechanisms that mediate Perk's effect on sumo targeting to RIDD are unclear. Interactions between translational regulation and the RIDD pathway are not unprecedented, as protection from translational attenuation is one way by which mRNAs at the ER membrane can escape degradation by RIDD [20]. It is possible that translation of the sumo transcript is especially attenuated when Perk is activated, or that its degradation is especially reliant on this attenuation, perhaps facilitating the formation of the sumo stem loop structure or allowing Ire1 greater access to the mRNA through ribosome depletion. It is also possible that translation attenuation is generally required for RIDD, but sumo is uniquely unaffected by Perk-independent mechanisms of attenuation that occur during ER stress in S2 cells [33]. Beyond attenuation of general translation, Perk-dependent eIF2α phosphorylation also enhances translation of certain mRNAs such as Atf4 and Gadd34 [8], [34]. It is unlikely that such a protein is mediating Perk's effect on sumo degradation, as sumo mRNA is still degraded during ER stress when translation is inhibited (Figure 1E). Because we have not specifically examined transcription of sumo in Perk-depleted cells, it is also possible that Perk knockdown indirectly affects sumo transcription by an unknown mechanism.

Overall, we propose that while ER localization is a key factor in targeting most mRNAs for RIDD in *D. melanogaster* cells, stable membrane association can be overcome by the presence of a specific Ire1 recognition site coupled with translational attenuation via Perk. Although this appears to be an exception to the general RIDD targeting rules in flies, this mechanism may be more prevalent in other organisms. RIDD targets in all systems studied so far are enriched for mRNAs that are predicted to localize to the ER [17], [18], [19], [35], but mammalian RIDD targets are also enriched for mRNAs containing Xbp1-like stem loops [18], [19], [22]. Interestingly, while preferred cleavage sites of several RIDD targets have been determined in both *D. melanogaster* and *S. pombe*, mutagenesis of these sites results in cleavage at alternative sites allowing for degradation to still occur [17], [21]. In contrast, mutagenesis of residues important for cleavage of at least two mammalian RIDD targets inhibits their degradation *in vitro* [22], [36]. These correlations suggest that while sumo regulation by RIDD does not appear to be widely conserved, the targeting mechanism exemplified by sumo may be more generally applicable to RIDD in other organisms, including mammals.

## Materials and Methods

### Cell culture, ER stress induction, and RNAi

We cultured *Drosophila* S2 cells (Invitrogen) at room temperature in Schneider's media (Invitrogen) supplemented with 10% fetal bovine serum and antibiotics. Unless otherwise indicated, we induced ER stress for 5 hours with 2 mM DTT.

For RNAi experiments, we amplified regions of the Ire1 (CG4583), Perk, and Xbp1 coding sequences (CDS) from S2 cell cDNA using primers containing T7 RNA polymerase sites on the 5′ ends. We then synthesized dsRNA from these templates using the Megascript T7 kit (Ambion). We incubated S2 cells with dsRNA in serum free media for 45 minutes, replaced the serum, and allowed cells to recover for 4 days. We then repeated the dsRNA treatment and subjected cells to ER stress one day following the second dsRNA treatment.

We cultured MC3T3-E1 (ATCC) and Hek293 (from A.V. Maricq lab) cells following ATCC guidelines in MEMα and DMEM media (Invitrogen), respectively, supplemented with 10% fetal bovine serum and antibiotics. For Ire1 knockdown experiments we used organism-specific Ire1 siRNA (Qiagen) and followed Invitrogen RNAimax guidelines for transfection of siRNA. We subjected cells to ER stress 48–72 hours after transfection, when cells were approximately 80% confluent, and collected RNA after 4 hours.

### Quantitative real-time PCR

For all RNA analyses, we isolated total RNA using Trizol reagent (Invitrogen), and synthesized cDNA using 2 µg of total RNA as a template, T18 as a primer, and M-MuLV reverse transcriptase (NEB). We measured relative mRNA abundance by real time quantitative PCR using a Mastercycler ep realplex (Eppendorf) with SYBR Green as the fluorescent dye. We measured each sample in triplicate and normalized to the ribosomal protein Rpl19 mRNA. To control for contaminating plasmid or genomic DNA we also measured samples to which no reverse transcriptase was added. The primers used for qPCR are given in Table 1.

**Table 1:** Primers used for qPCR.

Gene Name	Primer1	Primer 2
*Dm* sumo (smt3)	TTTGTTATTTACGCACACAGACG	GTCTGACGAAAAGAAGGGAGG
*Dm* Ribosomal Protein L19 (Rpl19)	AGGTCGGACTGCTTAGTGACC	CGCAAGCTTATCAAGGATGG
*Dm* Act5C (actin)	ATGTGTGACGAAGAAGTTGCT	GAAGCACTTGCGGTGCACAAT
*Dm* sparc	AAAATGGGCTGTGTCCTAACC	TGCAGCACAATCTACTCAATCC
*Dm* Xbp1	GGCCATCAACGAGTCACTGCT	TGTGTCCACCTGTTGTATACC
*Dm* Tsp42Ee	AACAACGTGCGTAACTACAAGC	TTCCAAATTTAAATCTTTCCCG
*Dm* CG3984	CTACTGTTGTTCCTGGTACCCC	CTGGTTGCTCAGTAACACTTGG
*Dm* Hydr2	CGCATACACGACTATTTAACGC	TTTGGTTTCTCTTTGATTTCCG
*Dm* CG6650	ACAATGGGACAGGCAAAGAC	GGTGACATTCGTTTCCGAGT
*Dm* sumo reporters	CAGTGCAACTAAAGGGGGGATC	TTTGTTATTTACGCACACAGACG, or TCCGTCGCGGCCGCTTATGGAGCGCCACCAGTCTGCT
GFP reporters	CCTGAAGTTCATCTGCACCA	TGCTCAGGTAGTGGTTGTCG
*Mm* Rpl19	CTGATCAAGGATGGGCTGAT	GCCGCTATGTACAGACACGA
*Mm and Hs* sumo1	GGAGGCAAAACCTTCAACTG	CCCCGTTTGTTCCTGATAAA
*Mm* sumo2	GGGAGCCTGCTACTTTACTCC	TCCATCTCATGTCAACCAGAA
*Mm* sumo3	GATGGCTCGGTGGTACAGTT	TGTCCTCATCCTCCATCTCC
*Mm and Hs* Blos1	CAAGGAGCTGCAGGAGAAGA	GCCTGGTTGAAGTTCTCCAC
*Hs* Rpl19	ATGTATCACAGCCTGTACCTG	TTCTTGGTCTCTTCCTCCTTG
*Hs* sumo2	AGCTGAGGAGACTCCGGCGCTCGC	AGTAGACACCTCCCGTCTGC
*Hs* sumo3	AGAATGACCACATCAACC	AGTAGACACCTCCCGTCTGC

Dm = Drosophila melanogaster. Mm = Mus musculus. Hs = Homo sapiens.

### Digitonin fractionation

To separate membrane and cytosolic mRNAs we used a method developed by Stephens and Nicchitta [23] and modified in our previous studies [20]. Briefly, we incubated S2 cells with or without DTT (2 mM, 20 minutes), added cycloheximide (35 µM) for 10 min, and collected cells by centrifugation. We then resuspended cells in cytosol buffer (150 mM KOAc, 20 mM Hepes pH 7.5, 2.5 mM Mg(OAc)_2_, 200 U/mL RNaseOUT, 35 µM cycloheximide) containing 1 mg/mL digitonin (15 min on ice). We then centrifuged the lysates (800 xg, 5 min at 4 C) and collected the supernatant as the cytosolic fraction. We resuspended the pellet in cytosol buffer with 1% Triton X-100 (15 min on ice), centrifuged as above and collected the supernatant as the membrane-bound fraction. We measured the abundance of specific RNAs in each fraction by qPCR and calculated the fraction membrane as the abundance of a particular mRNA in the membrane-bound fraction divided by the sum of that mRNA's abundance in the cytosolic and membrane-bound fractions.

### Plasmids and reporter RNA analyses

For sumo reporters, we amplified the sumo (smt3, CG4494) CDS from S2 cell cDNA and subcloned into an expression vector containing the copper-inducible *D. melanogaster* metallothionein promoter described previously [17]. To examine the effects of the 3′UTR, we separately amplified the 3′UTRs of sumo, sparc (CG6378), and Gapdh1 (CG12055) from S2 cDNA and subcloned into the sumo expression vector just downstream of the CDS. We introduced mutations into the sumo vector containing the sumo 3′UTR for Figure 2C using PCR-based mutagenesis. For GFP reporters, we used a previously-described EGFP reporter in the copper-inducible expression vector [20], and replaced the vector SV40 3′UTR with that from Gapdh1. To introduce the sumo SL, we added the 30 nucleotide sequence from the 3′ end of the sumo CDS (including the stop codon) in-frame to the 3′ end of the GFP CDS.

We generated stable, polyclonal cell lines by cotransfecting our expression plasmids (1.8 µg) with a puromycin resistance plasmid (0.2 µg) using Cellfectin II (Invitrogen) and selecting for resistant cells. To monitor decay of mRNAs expressed from reporter constructs, we treated cells with CuSO_4_ (200 µM overnight) to induce expression, collected “time 0” RNA samples, then washed cells to remove the CuSO_4_. We have previously shown this procedure to be effective in blocking transcription of the reporter mRNA, such that subsequent measurements reflect decay rates [20]. We then either left cells untreated or added DTT (2 mM) and collected RNA samples after 5 hours. RNA abundance measurements were normalized to the level of RNA in the CuSO_4_-treated cells.

### Western blot analysis

We washed cells in PBS before lysing in 1x RIPA buffer (25 mM Tris, pH 7.6, 150 mM NaCl, 1% NP-40, 1% Na- deoxycholate, and 0.1% SDS) with protease inhibitors (Thermo scientific). We resolved protein on NuPage Bis-Tris 4–12% gels (Invitrogen), transferred them to nitrocellulose membranes and probed for total eIF2α (abcam, 1∶500) or Ser51-P eIF2α (abcam 26197, 1∶1000) followed by a secondary IRDye 800CW antibody (Licor 926-32210, 1∶10000). We visualized immunoblots using a Licor Odyssey imager.

### Xbp1 splicing assay

Using S2 cDNA as a template we assayed Xbp1 splicing through PCR analysis of a fragment of the Xbp1 transcript encompassing the 23 nucleotide splice site. We resolved the spliced and unspliced products using a 2% agarose gel. Primers for this assay are shown in Table 1.

## References

[1] Moore KA, Hollien J (2012) The unfolded protein response in secretory cell function. Annu Rev Genet 46: 165–183. 22934644 10.1146/annurev-genet-110711-155644

[2] Walter P, Ron D (2011) The unfolded protein response: from stress pathway to homeostatic regulation. Science 334: 1081–1086. 22116877 10.1126/science.1209038

[3] Wang S, Kaufman RJ (2012) The impact of the unfolded protein response on human disease. J Cell Biol 197: 857–867. 22733998 10.1083/jcb.201110131 PMC3384412

[4] Harding HP, Zhang Y, Ron D (1999) Protein translation and folding are coupled by an endoplasmic-reticulum-resident kinase. Nature 397: 271–274. 9930704 10.1038/16729

[5] Shi Y, Vattem KM, Sood R, An J, Liang J, et al (1998) Identification and characterization of pancreatic eukaryotic initiation factor 2 alpha-subunit kinase, PEK, involved in translational control. Mol Cell Biol 18: 7499–7509. 9819435 10.1128/mcb.18.12.7499 PMC109330

[6] Sood R, Porter AC, Ma K, Quilliam LA, Wek RC (2000) Pancreatic eukaryotic initiation factor-2alpha kinase (PEK) homologues in humans, Drosophila melanogaster and Caenorhabditis elegans that mediate translational control in response to endoplasmic reticulum stress. Biochem J 346 Pt 2: 281–293. PMC1220852 10677345

[7] Harding HP, Zhang Y, Bertolotti A, Zeng H, Ron D (2000) Perk is essential for translational regulation and cell survival during the unfolded protein response. Mol Cell 5: 897–904. 10882126 10.1016/s1097-2765(00)80330-5

[8] Harding HP, Novoa I, Zhang Y, Zeng H, Wek R, et al.. (2000) Regulated translation initiation controls stress-induced gene expression in mammalian cells. Mol Cell. United States. pp. 1099–1108. 10.1016/s1097-2765(00)00108-8 11106749

[9] Credle JJ, Finer-Moore JS, Papa FR, Stroud RM, Walter P (2005) On the mechanism of sensing unfolded protein in the endoplasmic reticulum. Proc Natl Acad Sci U S A 102: 18773–18784. 16365312 10.1073/pnas.0509487102 PMC1316886

[10] Cox JS, Shamu CE, Walter P (1993) Transcriptional induction of genes encoding endoplasmic reticulum resident proteins requires a transmembrane protein kinase. Cell 73: 1197–1206. 8513503 10.1016/0092-8674(93)90648-a

[11] Mori K, Ma W, Gething MJ, Sambrook J (1993) A transmembrane protein with a cdc2+/CDC28-related kinase activity is required for signaling from the ER to the nucleus. Cell 74: 743–756. 8358794 10.1016/0092-8674(93)90521-q

[12] Calfon M, Zeng H, Urano F, Till JH, Hubbard SR, et al (2002) IRE1 couples endoplasmic reticulum load to secretory capacity by processing the XBP-1 mRNA. Nature 415: 92–96. 11780124 10.1038/415092a

[13] Yoshida H, Matsui T, Yamamoto A, Okada T, Mori K (2001) XBP1 mRNA is induced by ATF6 and spliced by IRE1 in response to ER stress to produce a highly active transcription factor. Cell. United States. pp. 881–891. 10.1016/s0092-8674(01)00611-0 11779464

[14] Haze K, Yoshida H, Yanagi H, Yura T, Mori K (1999) Mammalian transcription factor ATF6 is synthesized as a transmembrane protein and activated by proteolysis in response to endoplasmic reticulum stress. Mol Biol Cell 10: 3787–3799. 10564271 10.1091/mbc.10.11.3787 PMC25679

[15] Lee AH, Iwakoshi NN, Glimcher LH (2003) XBP-1 regulates a subset of endoplasmic reticulum resident chaperone genes in the unfolded protein response. Mol Cell Biol 23: 7448–7459. 14559994 10.1128/MCB.23.21.7448-7459.2003 PMC207643

[16] Lu PD, Harding HP, Ron D (2004) Translation reinitiation at alternative open reading frames regulates gene expression in an integrated stress response. J Cell Biol. United States. pp. 27–33. 10.1083/jcb.200408003 PMC2172506 15479734

[17] Hollien J, Weissman JS (2006) Decay of endoplasmic reticulum-localized mRNAs during the unfolded protein response. Science 313: 104–107. 16825573 10.1126/science.1129631

[18] Hollien J, Lin JH, Li H, Stevens N, Walter P, et al (2009) Regulated Ire1-dependent decay of messenger RNAs in mammalian cells. J Cell Biol 186: 323–331. 19651891 10.1083/jcb.200903014 PMC2728407

[19] Han D, Lerner AG, Vande Walle L, Upton JP, Xu W, et al.. (2009) IRE1alpha kinase activation modes control alternate endoribonuclease outputs to determine divergent cell fates. Cell. United States. pp. 562–575. 10.1016/j.cell.2009.07.017 PMC2762408 19665977

[20] Gaddam D, Stevens N, Hollien J (2013) Comparison of mRNA localization and regulation during endoplasmic reticulum stress in Drosophila cells. Mol Biol Cell 24: 14–20. 23135994 10.1091/mbc.E12-06-0491 PMC3530775

[21] Kimmig P, Diaz M, Zheng J, Williams CC, Lang A, et al (2012) The unfolded protein response in fission yeast modulates stability of select mRNAs to maintain protein homeostasis. elife 1: e00048. 23066505 10.7554/eLife.00048 PMC3470409

[22] Hur KY, So JS, Ruda V, Frank-Kamenetsky M, Fitzgerald K, et al.. (2012) IRE1alpha activation protects mice against acetaminophen-induced hepatotoxicity. J Exp Med. United States. pp. 307–318. 10.1084/jem.20111298 PMC3280871 22291093

[23] Stephens SB, Dodd RD, Lerner RS, Pyhtila BM, Nicchitta CV (2008) Analysis of mRNA partitioning between the cytosol and endoplasmic reticulum compartments of mammalian cells. Methods Mol Biol 419: 197–214. 18369985 10.1007/978-1-59745-033-1_14

[24] Calfon M, Zeng H, Urano F, Till JH, Hubbard SR, et al (2002) IRE1 couples endoplasmic reticulum load to secretory capacity by processing the XBP-1 mRNA. Nature 415: 92–96. 11780124 10.1038/415092a

[25] Kawai T, Fan J, Mazan-Mamczarz K, Gorospe M (2004) Global mRNA stabilization preferentially linked to translational repression during the endoplasmic reticulum stress response. Mol Cell Biol. United States. pp. 6773–6787. 10.1128/MCB.24.15.6773-6787.2004 PMC444849 15254244

[26] Majumder M, Huang C, Snider MD, Komar AA, Tanaka J, et al.. (2012) A novel feedback loop regulates the response to endoplasmic reticulum stress via the cooperation of cytoplasmic splicing and mRNA translation. Mol Cell Biol. United States. pp. 992–1003. 10.1128/MCB.06665-11 PMC3295193 22215619

[27] Kawahara T, Yanagi H, Yura T, Mori K (1998) Unconventional splicing of HAC1/ERN4 mRNA required for the unfolded protein response. Sequence-specific and non-sequential cleavage of the splice sites. J Biol Chem 273: 1802–1807. 9430730 10.1074/jbc.273.3.1802

[28] Iwakoshi NN, Lee AH, Vallabhajosyula P, Otipoby KL, Rajewsky K, et al (2003) Plasma cell differentiation and the unfolded protein response intersect at the transcription factor XBP-1. Nat Immunol 4: 321–329. 12612580 10.1038/ni907

[29] Gonzalez TN, Sidrauski C, Dorfler S, Walter P (1999) Mechanism of non-spliceosomal mRNA splicing in the unfolded protein response pathway. Embo j 18: 3119–3132. 10357823 10.1093/emboj/18.11.3119 PMC1171393

[30] Flotho A, Melchior F (2013) Sumoylation: a regulatory protein modification in health and disease. Annu Rev Biochem 82: 357–385. 23746258 10.1146/annurev-biochem-061909-093311

[31] Chen H, Qi L (2010) SUMO modification regulates the transcriptional activity of XBP1. Biochem J 429: 95–102. 20408817 10.1042/BJ20100193 PMC2964647

[32] Jiang Z, Fan Q, Zhang Z, Zou Y, Cai R, et al (2012) SENP1 deficiency promotes ER stress-induced apoptosis by increasing XBP1 SUMOylation. Cell Cycle 11: 1118–1122. 22370484 10.4161/cc.11.6.19529

[33] Garrey JL, Lee YY, Au HH, Bushell M, Jan E (2010) Host and viral translational mechanisms during cricket paralysis virus infection. J Virol 84: 1124–1138. 19889774 10.1128/JVI.02006-09 PMC2798387

[34] Lee YY, Cevallos RC, Jan E (2009) An upstream open reading frame regulates translation of GADD34 during cellular stresses that induce eIF2alpha phosphorylation. J Biol Chem 284: 6661–6673. 19131336 10.1074/jbc.M806735200 PMC2652341

[35] Kimmig P, Diaz M, Zheng J, Williams CC, Lang A, et al.. (2012) The unfolded protein response in fission yeast modulates stability of select mRNAs to maintain protein homeostasis. Elife. United States. pp. e00048. 10.7554/eLife.00048 PMC3470409 23066505

[36] So JS, Hur KY, Tarrio M, Ruda V, Frank-Kamenetsky M, et al (2012) Silencing of lipid metabolism genes through IRE1alpha-mediated mRNA decay lowers plasma lipids in mice. Cell Metab 16: 487–499. 23040070 10.1016/j.cmet.2012.09.004 PMC3475419

